# Adverse events of romidepsin versus tucidinostat for peripheral T-cell lymphoma: a pharmacovigilance study using the Japanese adverse drug event report database

**DOI:** 10.1186/s40780-026-00565-3

**Published:** 2026-03-27

**Authors:** Nao Takatsu, Jun Matsumoto, Yurie Oka, Tomonori Sakai, Naohiro Iwata, Tsukasa Higashionna, Tatsuaki Takeda, Hirofumi Hamano, Yoshito Zamami

**Affiliations:** 1 Department of Pharmacy, Okayama University Hospital, Okayama, Japan; 2 Department of Personalised Medicine and Preventive Healthcare Sciences, Faculty of Medicine, Dentistry and Pharmaceutical Sciences, Okayama University, 2-5-1 Shikata, Kita-ku, Okayama, Japan; 3 Department of Clinical Pharmacology, Faculty of Medicine, Dentistry and Pharmaceutical Sciences, Okayama University, Okayama, Japan; 4 Department of Education and Research Centre for Clinical Pharmacy, Faculty of Pharmaceutical Sciences, Okayama University, Okayama, Japan; 5 Department of Pharmacy, Medical Development Field, Okayama University, Okayama, Japan

**Keywords:** Romidepsin, Tucidinostat, Adverse event, JADER, Pharmacovigilance

## Abstract

**Background:**

Peripheral T-cell lymphoma (PTCL) is a heterogeneous group of lymphomas with poor prognosis, particularly in patients with relapsed or refractory (R/R) disease. Romidepsin and tucidinostat are histone deacetylase inhibitors used to treat R/R PTCL. No head-to-head post-marketing surveillance studies have compared adverse events (AEs) between the two agents. In this brief report, the AE profiles of romidepsin and tucidinostat were compared using the Japanese Adverse Drug Event Report (JADER) database to facilitate their differentiation and promote the management of AEs.

**Methods:**

We conducted a descriptive analysis using data from the JADER database from April 2018 to July 2025. The reported AEs for romidepsin and tucidinostat were extracted and classified according to preferred terms (PTs) and system organ classes (SOCs). Reporting odds ratios with 95% confidence intervals were calculated to compare the AE profiles between the groups.

**Results:**

In total, 998,397 reports were analysed for all drugs, including 323 for romidepsin and 753 for tucidinostat. Compared with all drugs, both agents showed significant disproportionality signals in four SOCs: Blood and lymphatic system disorders; General disorders and administration site conditions; Investigations; and Neoplasms benign, malignant and unspecified. Romidepsin exhibited additional significant signals in six SOCs: Cardiac disorders, Eye disorders, Gastrointestinal disorders, Immune system disorders, Infections and infestations, and Metabolism and nutrition disorders. Direct comparison between the two agents revealed broader AE profiles for romidepsin, with AEs more frequently reported in eight SOCs, whereas tucidinostat showed AEs in only two SOCs. Romidepsin was associated with AEs more frequently reported in several PTs, including atrial fibrillation and gastrointestinal toxicities, such as constipation, tumour lysis syndrome, hepatotoxicity, and peripheral neuropathy, which was consistent with the results at the SOC level. In contrast, several significant PTs for tucidinostat were observed in General disorders and administration site conditions and Investigations.

**Conclusions:**

The Japanese real-world pharmacovigilance analysis showed differences in the AE profiles between romidepsin and tucidinostat. These differences in safety profiles may be useful for treatment selection and AE management in routine clinical practice among patients with R/R PTCL. Further studies are warranted to confirm these findings and better characterise the safety profiles of these agents.

**Supplementary information:**

The online version contains supplementary material available at 10.1186/s40780-026-00565-3.

## Background

Peripheral T-cell lymphoma (PTCL) is a heterogeneous group of lymphomas derived from post-thymic lymphocytes [1, 2]. PTCL is more prevalent in Asia, including Japan, where it accounts for approximately 15–20% of all lymphomas [3], compared with approximately 5–10% in Western countries such as the United States and the United Kingdom [4, 5]. The prognosis of PTCL remains poor, with a 5-year overall survival rate of approximately 40–50%, although outcomes vary depending on the disease subtype and the use of stem cell transplantation [5]. In particular, patients with relapsed/refractory (R/R) PTCL experience worse outcomes, with a 5-year overall survival rate of only 10–20% without stem cell transplantation [6, 7]. Thus, substantial challenges remain in the treatment of PTCL, particularly in the R/R setting.

Romidepsin and tucidinostat (also known as chidamide) are histone deacetylase inhibitors used in patients with R/R PTCL [8, 9]. Regulatory approval for these agents varies across countries. Romidepsin was approved in the United States in 2011 but not in China or the United Kingdom, whereas tucidinostat was approved in China in 2014 but not in the United States or the United Kingdom [10]. In contrast, Japan approved both agents—romidepsin in 2017 and tucidinostat in 2021. This regulatory landscape allows clinicians to select appropriate agents for patients with R/R PTCL in Japan. Nevertheless, there are no international guidelines encompassing both romidepsin and tucidinostat, as most countries have not approved both agents [10, 11]. Consequently, optimal criteria for their use remain unclear.

In addition to therapeutic efficacy, consideration of adverse events (AEs) is important, as they substantially influence treatment continuity and clinical outcomes. A recent meta-analysis revealed no significant differences in response rates between romidepsin and tucidinostat [12], thereby emphasising the importance of AE profiles in distinguishing these agents. In this brief report, we conducted a descriptive comparison of the AE profiles of romidepsin and tucidinostat using data from the Japanese Adverse Drug Event Report (JADER) database, a national pharmacovigilance database in Japan [13], where both agents have been approved.

## Materials and methods

### Data processing

Datasets for romidepsin and tucidinostat, covering the period from April 2018 (when romidepsin was launched in Japan) to July 2025, were extracted from the JADER database (Supplementary Figure 1). All AEs coded as preferred terms (PTs) were extracted and assigned to primary system organ classes (SOCs) according to the Medical Dictionary for Regulatory Activities, version 28.1 [14]. The extraction process was independently conducted by two authors to ensure reproducibility. The requirement for institutional review board approval was waived because all patient information was completely anonymised. This study was conducted in accordance with global pharmacovigilance guidelines and with the recommended checklist for analyses using JADER [15–17].

### Reporting odds ratio calculation

Analyses using the reporting odds ratio (ROR) were conducted to compare the numbers of PTs and SOCs between the groups. In this study, RORs were calculated either using all other drugs to assess disproportionality or the other drug for direct comparison between the two drugs, as described previously in other studies and ours (Supplementary Table 1) [18–20]. Zero cells were corrected using a continuity correction of 0.5 applied to all cells (Haldane–Anscombe correction). The proportional reporting ratio was not applied because it yields results mathematically similar to those of the ROR. Bayesian-based approaches were also not applied owing to the risk of false negatives with small sample sizes. Consequently, the ROR was considered an appropriate method for this study, maintaining methodological simplicity and consistency.

### Statistical analysis

Categorical variables were compared between the two groups using Fisher’s exact test. All analyses were conducted when either group had more than three reported cases. Statistical significance was evaluated based on the thresholds of the EudraVigilance criteria [21]. For comparisons with all drugs, a significant signal was defined as an ROR exceeding 1.0, with the lower limit of 95% CI > 1.0. For comparisons between romidepsin and tucidinostat, significance was defined as an ROR > 1.0 or <1.0 with a 95% CI that did not overlap 1.0. All statistical analyses were performed using JMP, version 15 (SAS Institute Inc., Cary, NC, USA).

## Results

### Comparison of AE profiles for romidepsin and tucidinostat with all drugs

A total of 998,397 reports were extracted for all drugs, including 323 for romidepsin and 753 for tucidinostat (Supplementary Figure 1). Overall, 176 and 188 PTs were identified for romidepsin and tucidinostat, respectively. After consolidating overlapping PTs between the two agents, 281 unique PTs across 22 SOCs were included. Demographic and reporting characteristics are shown in Table 1.

**Table 1:** Demographic and characteristics of the study population

Subject		Romidepsin		Tucidinostat	
		*n*	%	*n*	%
Total		323	100.0	753	100.0
Age					
	<70	105	32.5	197	26.2
	≥70	209	64.7	543	72.1
	Unknown	9	2.8	13	1.7
Sex					
	Male	200	61.9	387	51.4
	Female	115	35.6	308	40.9
	Unknown	8	2.5	58	7.7
Reporting year					
	2018	62	19.2	0	0.0
	2019	99	30.7	0	0.0
	2020	40	12.4	0	0.0
	2021	26	8.0	12	1.6
	2022	60	18.6	195	25.9
	2023	24	7.4	319	42.4
	2024	11	3.4	189	25.1
	2025	1	0.3	38	5.0
Reporter type					
	Physician	283	87.6	591	78.5
	Pharmacist	23	7.1	14	1.9
	Other health professional	9	2.8	0	0.0
	Consumer	1	0.3	0	0.0
	Others^*^	7	2.2	148	19.7

*Reports submitted by multiple reporters rather than by a single healthcare professional

**Fig. 1:**
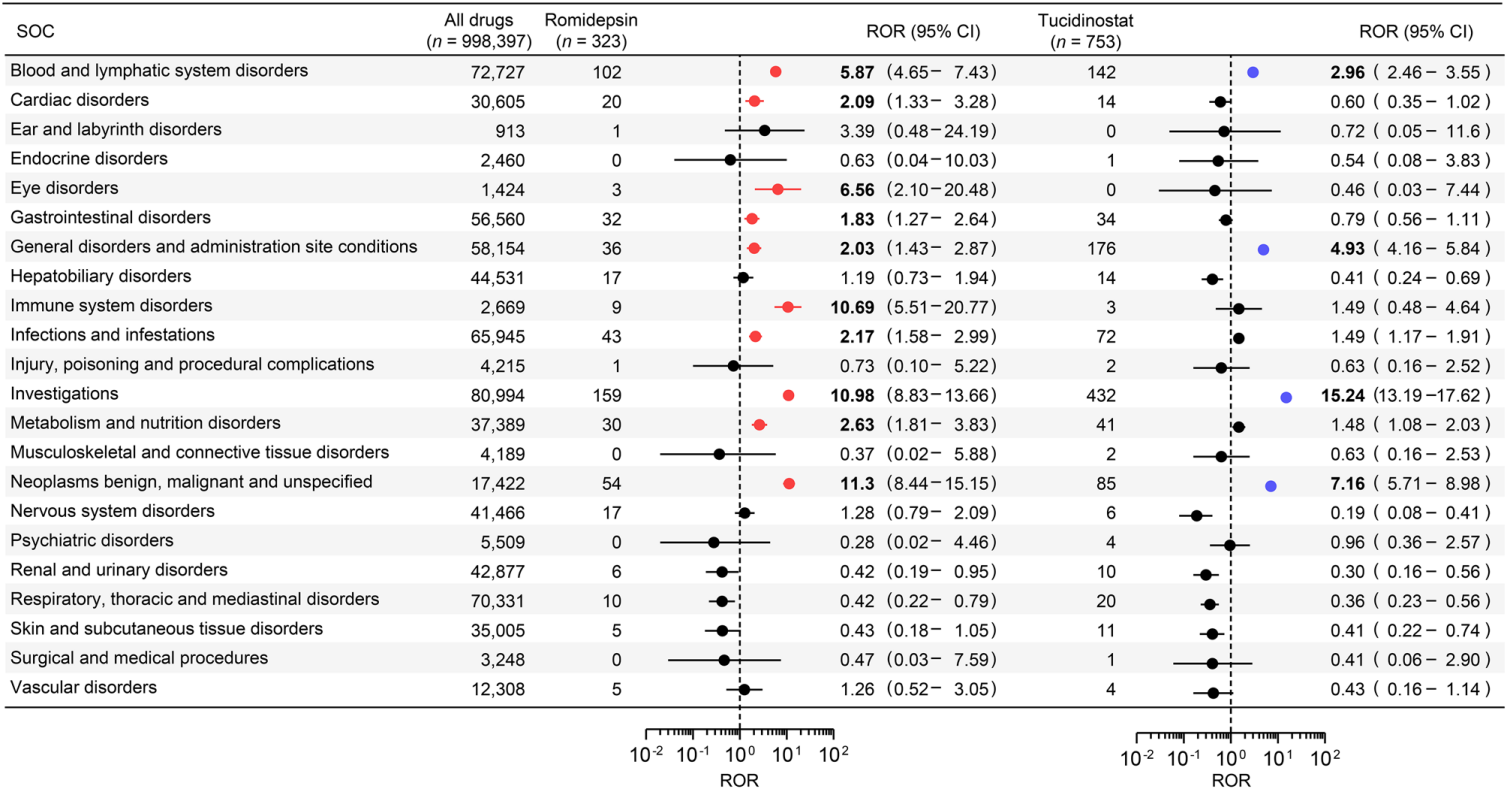
Disproportionality analysis of adverse events at the system organ class (SOC) level for romidepsin and tucidinostat compared with all drugs red and blue circles indicate reporting odds ratios (RORs) significantly greater than 1.0, with the lower limit of the 95% confidence interval (CI) >1.0, for romidepsin and tucidinostat, respectively. Both axes are plotted on a logarithmic scale

To characterise the AE profiles of romidepsin and tucidinostat, AEs associated with these agents were compared with those reported for all drugs at the SOC level (Fig. 1). For both agents, significant signals were observed for four SOCs: Blood and lymphatic system disorders; General disorders and administration site conditions; Investigations; and Neoplasms benign, malignant and unspecified (ROR > 1.0, *p* < 0.05). Furthermore, significant signals were observed only for romidepsin in six SOCs: Cardiac disorders (ROR [95% CI]: 2.09 [1.33–3.28]), Eye disorders (6.56 [2.10–20.48]), Gastrointestinal disorders (1.83 [1.27–2.64]), Immune system disorders (10.69 [5.51–20.77]), Infection and infestations (2.17 [1.58–2.99]), and Metabolism and nutrition disorders (2.63 [1.81–3.83]).

### Differences in AE profiles of romidepsin and tucidinostat

**Fig. 2:**
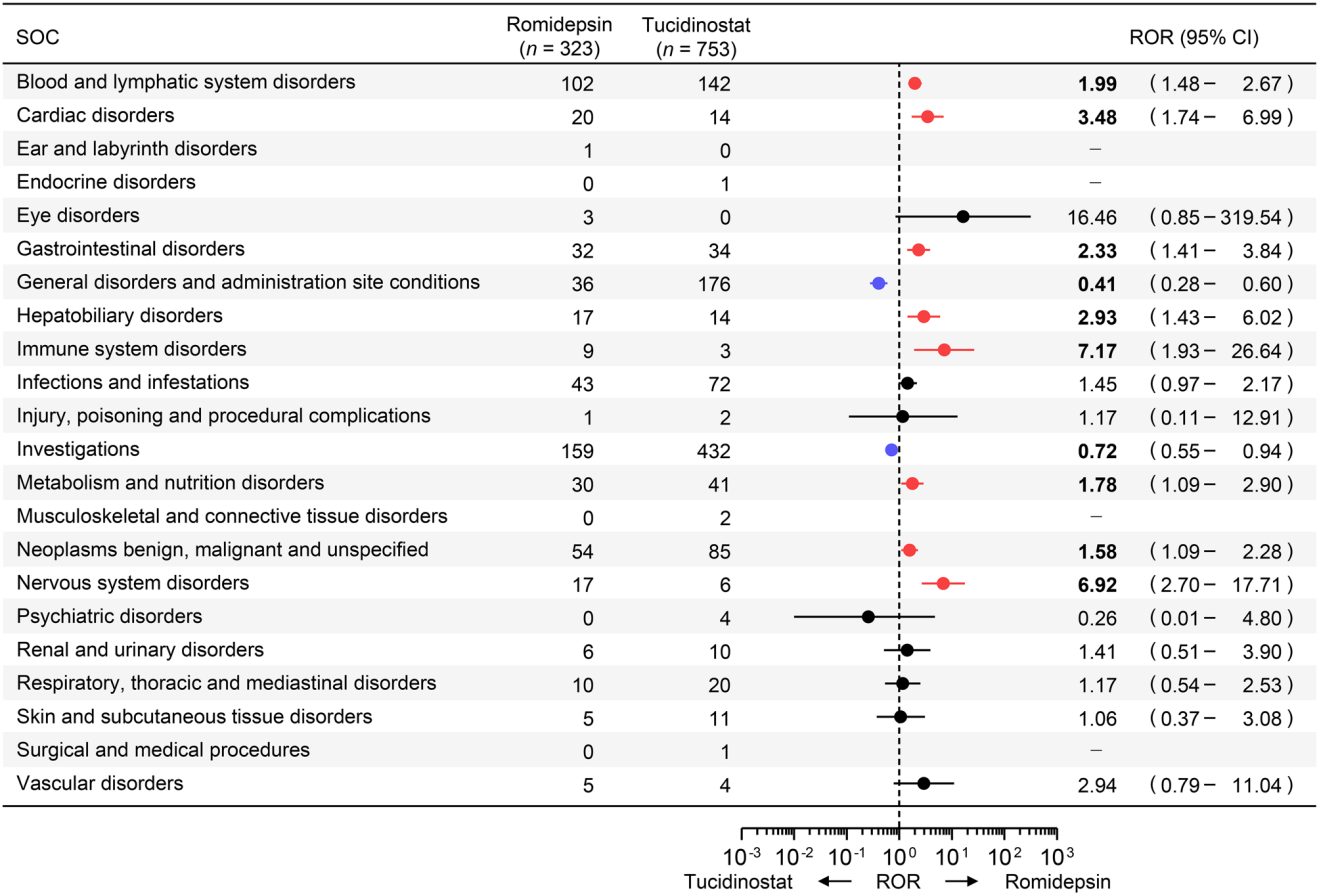
Disproportionality of adverse events between romidepsin and tucidinostat at the system organ class (SOC) level Red and blue circles indicate reporting odds ratios (RORs) significantly greater or lower than 1.0, respectively, with 95% confidence intervals (CIs) not overlapping 1.0, for romidepsin and tucidinostat, respectively. RORs were calculated using romidepsin as the reference for each comparison. The axis is plotted on a logarithmic scale

Differences in AE profiles between romidepsin and tucidinostat were assessed at the SOC level (Fig. 2). Romidepsin exhibited a broader AE profile, with AEs more frequently reported in eight SOCs: Blood and lymphatic system disorders (ROR [95% CI]: 1.99 [1.48–2.67]), Cardiac disorders (3.48 [1.74–6.99]), Gastrointestinal disorders (2.33 [1.41–3.84]), Hepatobiliary disorders (2.93 [1.43–6.02]), Immune system disorders (7.17 [1.93–26.64]), Metabolism and nutrition disorders (1.78 [1.09–2.90]), Neoplasms benign, malignant and unspecified (1.58 [1.09–2.28]), and Nervous system disorders (6.92 [2.70–17.71]). Conversely, only two SOCs were more frequently reported for tucidinostat: General disorders and administration site conditions (0.41 [0.28–0.60]) and Investigations (0.72 [0.55–0.94]).

**Fig. 3:**
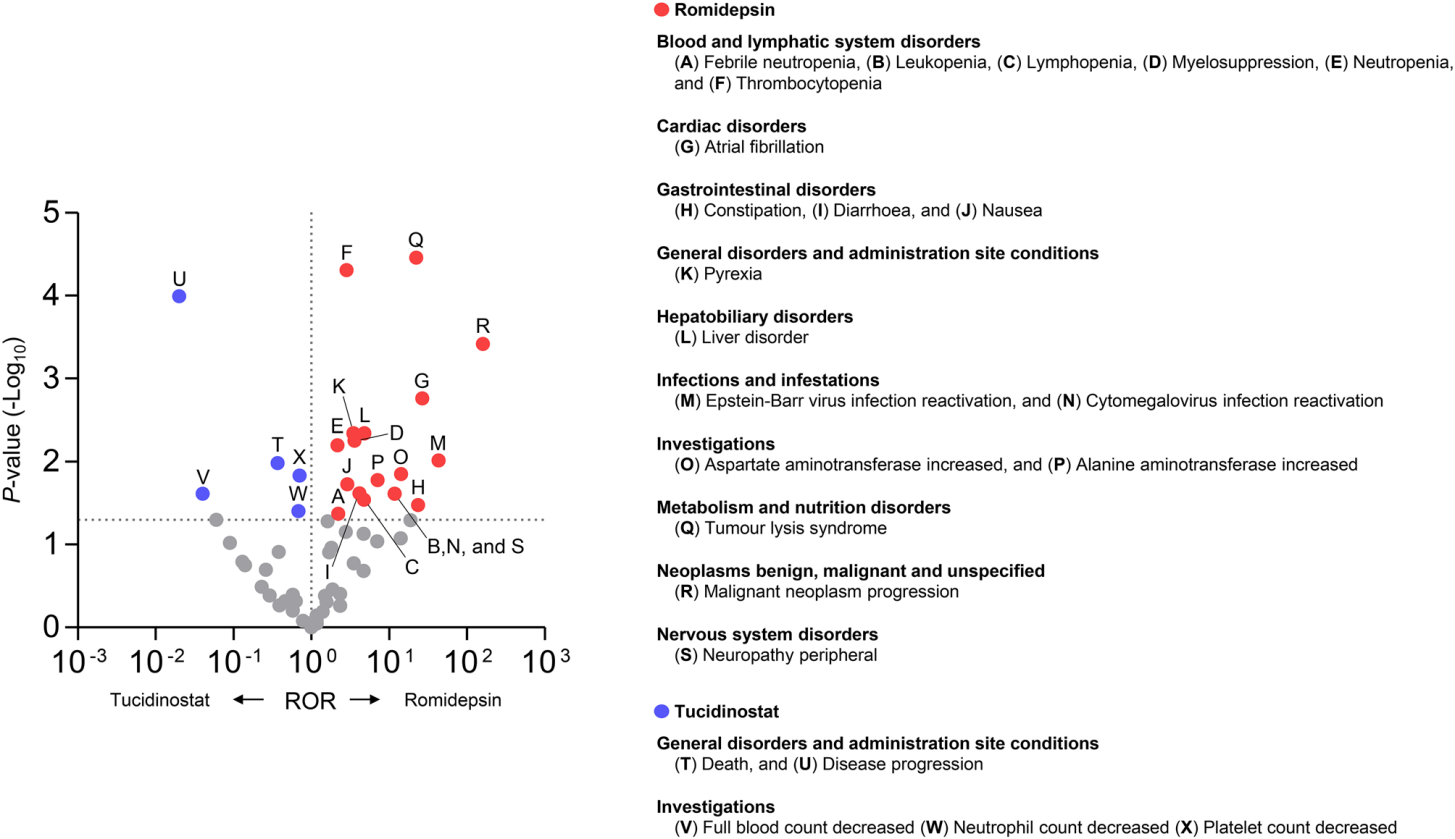
Identification of specific adverse events in comparison between romidepsin and tucidinostat at the preferred term (PT) level. Red and blue circles indicate reporting odds ratios (RORs) significantly greater or lower than 1.0, respectively, with 95% confidence intervals (CIs) not overlapping 1.0, for romidepsin and tucidinostat, respectively. The RORs were calculated using romidepsin as the reference for each comparison. The dashed lines indicate a *p*-value of 0.05 on the y-axis and an ROR of 1 on the x-axis. Both axes are plotted on a logarithmic scale

To identify specific AEs within the SOCs, the PTs of romidepsin and tucidinostat were compared. Most of the top 10 PTs for each agent were associated with SOCs that exhibited significant disproportionality in previous analyses (Supplementary Figure 2). Figure 3 presents all PTs that were reported significantly more frequently for romidepsin or tucidinostat. Consistent with the results at the SOC level, most PTs reported more frequently for romidepsin were categorised within SOCs that were also reported more frequently, including atrial fibrillation (26.51 [3.41–206.24]) in Cardiac disorders, constipation (26.62 [1.29–433.64]) in Gastrointestinal disorders, liver disorder in Hepatobiliary disorders (4.78 [1.62–14.10]), tumour lysis syndrome (22.16 [5.11–96.09]) in Metabolism and nutrition disorders, and neuropathy peripheral in Nervous system disorders (11.82 [1.38–101.62]). Conversely, several PTs for romidepsin showed significant or non-significant results, regardless of whether significance was observed at the corresponding SOC level. For tucidinostat, five PTs categorised within General disorders and administration site conditions and Investigations were reported significantly more frequently, consistent with the SOC-level findings.

## Discussion

This study compared the AE profiles of romidepsin and tucidinostat. Although primarily used as second- or later-line therapies, these agents remain important options for patients with PTCL who relapse and become refractory to or are intolerant to prior therapies owing to AEs. Moreover, recent clinical trials evaluating novel combination therapies involving these agents suggest their potential importance in future therapeutic strategies [22, 23].

Our results suggest that romidepsin may cause a higher frequency of AEs than tucidinostat in routine clinical practice, a trend also suggested by manufacturer-reported safety information, although these data are derived from separate clinical trials for each agent rather than direct comparative studies [24, 25]. In particular, atrial fibrillation (cardiac toxicity) and constipation, diarrhoea, and nausea (gastrointestinal toxicities) were identified as distinctive AEs of romidepsin, with substantial disproportionality and high reporting frequency compared to both all drugs and tucidinostat. In addition, given the significant disproportionality and high reporting frequency for hepatotoxicity and peripheral neuropathy with romidepsin, these AEs should be carefully considered when selecting romidepsin over tucidinostat.

Several PTs potentially related to Blood and lymphatic system disorders were identified and categorised under Investigations. Although both our findings and manufacturer-reported safety information suggest that haematotoxicity may occur more frequently with romidepsin than with tucidinostat [24, 25], the haematotoxicity associated with tucidinostat should not be overlooked. The reasons for the significant PTs observed for tucidinostat in General disorders and administration site conditions remain unclear. This SOC includes the PT term ‘disease progression’, which may reflect treatment efficacy rather than toxicity. Although a previous meta-analysis reported no significant difference in efficacy between romidepsin and tucidinostat [12], this analysis was based on clinical trial data obtained under controlled conditions with relatively homogeneous patient populations, whereas our study utilised a large-scale spontaneous reporting database that reflects real-world clinical practice and a more heterogeneous population. In addition, the efficacy of tucidinostat, but not romidepsin, varies substantially across PTCL subtypes, with higher efficacy observed in specific subtypes, such as angioimmunoblastic T-cell lymphoma [7, 8]. This may partly explain the discrepancy between the meta-analysis and our results; however, further investigation is required.

This study has several limitations inherent to analyses using spontaneous reporting databases, including reporting bias and the inability to determine incidence rates and risks. For example, this study analysed post-marketing data for romidepsin in Japan, whereas tucidinostat was launched in 2021, which may introduce potential bias owing to differences in reporting periods. Moreover, this study calculated RORs using not only all other drugs but also the other drug. Because the exact denominators of the data obtained from spontaneous reporting databases cannot be determined, the analysis does not provide a direct comparison of incidence or causal risk. Rather, it may reflect differences in AE reporting patterns between the two drugs, although it may be possible to estimate which AEs are more likely to be observed. Therefore, the results of the present study should be interpreted with caution.

Another limitation is that this study was based on a limited number of datasets, as patients with R/R PTCL are relatively few, and the JADER database is domestic. This may be related to inconsistencies between SOC- and PT-level analyses, where significant differences were observed at the SOC level but not at the PT level, as seen with Immune system disorders. Moreover, the database provides limited information on the severity of individual AEs and treatment efficacy. Although significant differences in reported frequencies were observed for the PTs ‘death’, ‘tumour lysis syndrome’, and ‘malignant neoplasm progression’, as well as ‘disease progression’ for tucidinostat, the implications require further investigation. Furthermore, analysing the associations between AEs and concomitant medications is challenging because information on concomitant drug use and timing is limited. Nevertheless, both romidepsin and tucidinostat are generally used as monotherapies in Japan, and their treatment lines are similar, although the potential influence of medications used by patients with other diseases cannot be excluded. Despite these limitations, this study is the first to demonstrate the differences in AE profiles between romidepsin and tucidinostat using real-world Japanese data.

## Conclusions

In this study, we compared the AE profiles of romidepsin and tucidinostat using the JADER database to support differentiation of their clinical use and AE management. We recommend that the findings of the present study be considered alongside therapeutic efficacy when selecting romidepsin or tucidinostat for patients with R/R PTCL.

## Electronic supplementary material

Below is the link to the electronic supplementary material.

Supplementary Material 1

## Data Availability

The datasets used and/or analysed in the current study are available from the corresponding author upon reasonable request.

## Abbreviations

CI: Confidence interval
JADER: Japanese Adverse Drug Event Report
PT: preferred term
PTCL: Peripheral T-cell lymphoma
ROR: Reporting odds ratio
R/R: Relapsed/refractory
SOC: System organ class.
