# Supplementary material for: Adverse events of romidepsin versus tucidinostat for peripheral T-cell lymphoma: a pharmacovigilance study using the Japanese adverse drug event report database
Source: J Pharm Health Care Sci. 2026 Mar 27;12:48. doi: 10.1186/s40780-026-00565-3 (PMC13151072; doi:10.1186/s40780-026-00565-3)
Supplement: Supplementary file 1 — Supplementary Material 1 [file 40780_2026_565_MOESM1_ESM.docx]

***Supplementary Materials***

**Adverse events of romidepsin versus tucidinostat for peripheral T-cell lymphoma: A pharmacovigilance study using the Japanese Adverse Drug Event Report database**

Nao Takatsu^1^, Jun Matsumoto^1,2*^, Yurie Oka^1,2^, Tomonori Sakai^1,3^, Naohiro Iwata^1^, Tsukasa Higashionna^1^, Tatsuaki Takeda^1,4^, Hirofumi Hamano^1,3^, and Yoshito Zamami^1,3^

^1^Department of Pharmacy, Okayama University Hospital, Okayama, Japan

^2^Department of Personalised Medicine and Preventive Healthcare Sciences, Faculty of Medicine, Dentistry and Pharmaceutical Sciences, Okayama University, Okayama, Japan

^3^Department of Clinical Pharmacology, Faculty of Medicine, Dentistry, and Pharmaceutical Sciences, Okayama University, Okayama, Japan

^4^Department of Education and Research Centre for Clinical Pharmacy, Faculty of Pharmaceutical Sciences, Okayama University, Okayama, Japan

*Corresponding author: Jun Matsumoto, Department of Personalized Medicine and Preventive Healthcare Sciences, Faculty of Medicine, Dentistry, and Pharmaceutical Sciences, Okayama University, 2-5-1 Shikata, Kita-ku, Okayama 700-8558, Japan; [matsumotoj@okayama-u.ac.jp](mailto:matsumotoj@okayama-u.ac.jp)

**Supplementary Table 1.** Cross-table methodology for calculation of reporting odds ratios based on the number of reports of adverse events (AEs) in this study.

| 2×2 contingency table^*^ | Number of reports with an AE of interest | Number of reports with other AEs or no AEs |
| --- | --- | --- |
| Drug of interest | a | b |
| All drugs or comparator | c | d |

^*^The ROR with a 95% CI was calculated as follows:

$$ROR=\frac{\frac{a}{b}}{\frac{c}{d}}$$

$$95\% CI=exp\{ln(ROR)\pm1.96\sqrt{\frac{1}{a}+\frac{1}{b}+\frac{1}{c}+\frac{1}{d}}\}$$

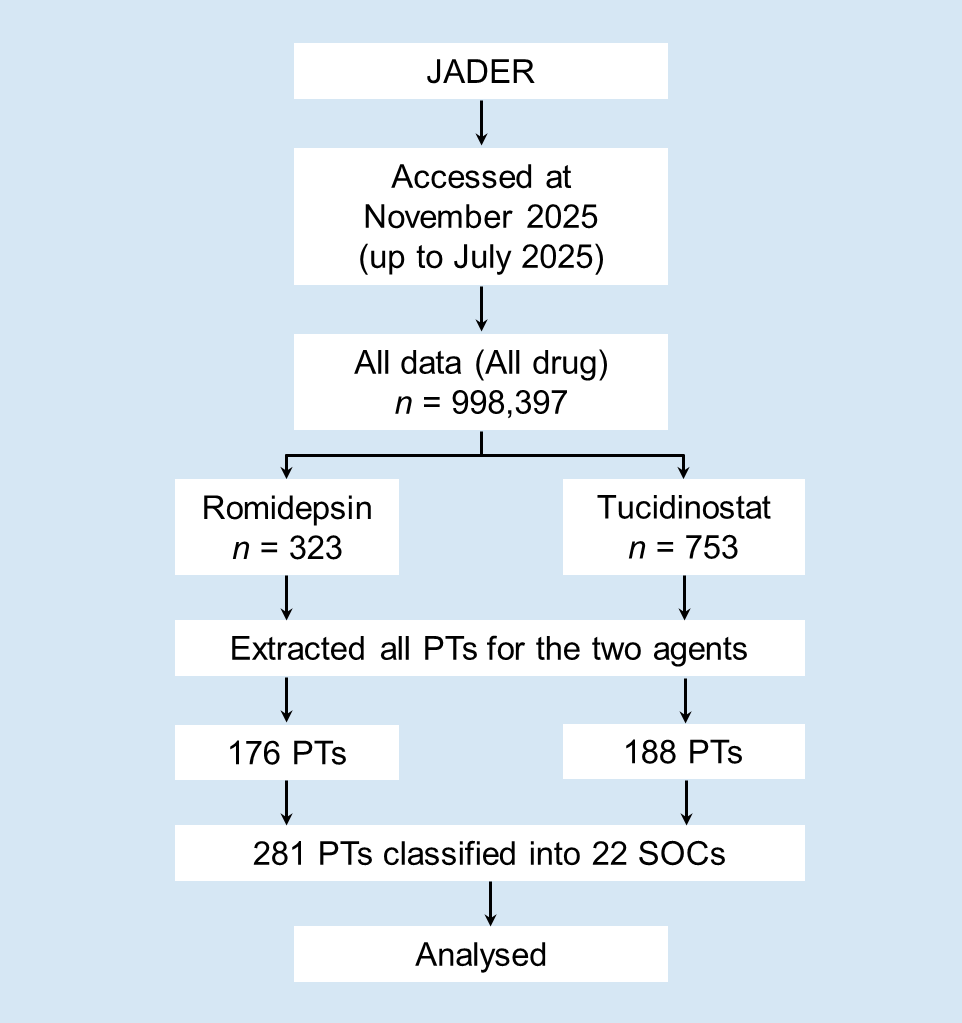


**Supplementary Figure 1.** Flow chart of the data extraction process from the Japanese Adverse Drug Event Report (JADER) database

PT, Preferred term; SOC, System organ class

**
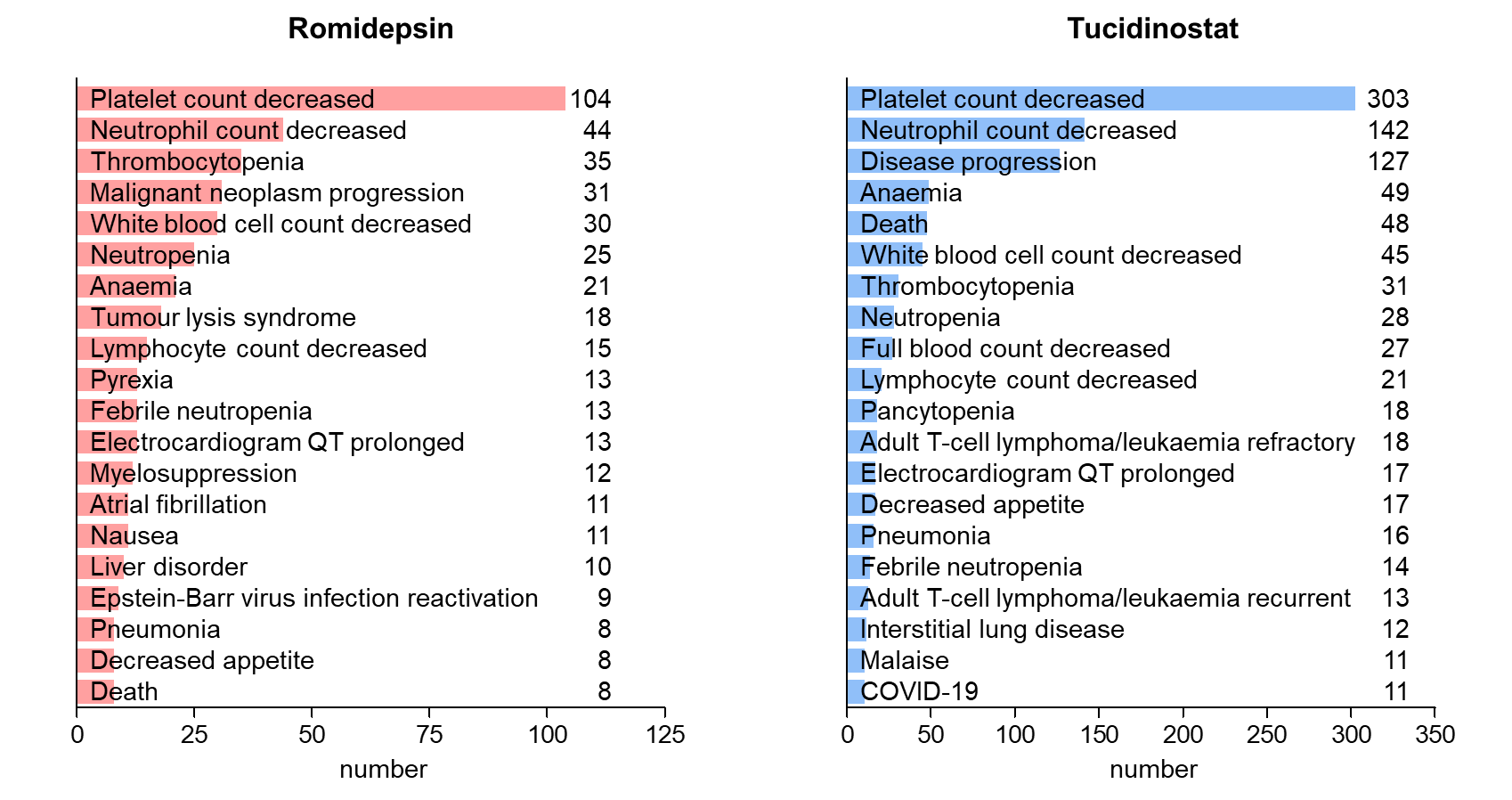
**

**Supplementary Figure 2.** Top 10 preferred terms (PTs) for romidepsin and tucidinostat

The axis indicates the number of reports.
